# The prognostic value of markers of right ventricular dysfunction in pulmonary embolism: a meta-analysis

**DOI:** 10.1186/cc10119

**Published:** 2011-03-28

**Authors:** Guillaume Coutance, Emmanuelle Cauderlier, Javed Ehtisham, Michèle Hamon, Martial Hamon

**Affiliations:** 1 Cardiologie, Centre Hospitalier Universitaire de Caen, Avenue Côte de Nacre, 14033 Caen, Normandy, France; 2 Service de radiologie, Centre Hospitalier Universitaire de Caen, Avenue Côte de Nacre, 14033 Caen, Normandy, France; 3 Inserm 744, Institut Pasteur de Lille, 1 rue du Professeur Calmette, 59019 Lille cedex, France

## Abstract

**Introduction:**

In pulmonary embolism (PE) without hemodynamic compromise, the prognostic value of right ventricular (RV) dysfunction as measured by echocardiography, computed tomography (CT) or biological (natriuretic peptides) markers has only been assessed in small studies.

**Methods:**

Databases were searched using the combined medical subject headings for right ventricular dysfunction or right ventricular dilatation with the exploded term acute pulmonary embolism. This retrieved 8 echocardiographic marker based studies (*n *= 1249), three CT marker based studies (*n *= 503) and 7 natriuretic peptide based studies (*n *= 582). A meta-analysis of these data was performed with the primary endpoint of mortality within three months after pulmonary embolism, and a secondary endpoint of overall mortality and morbidity by pulmonary embolism.

**Results:**

Patients with PE without hemodynamic compromise on admission and the presence of RV dysfunction determined by echocardiography and biological markers were associated with increased short-term mortality (odds ratio (OR) _ECHO _= 2.36; 95% confidence interval (CI): 1.3-43; OR _BNP _= 7.7; 95% CI: 2.9-20) while CT was not (OR_CT _= 1.54-95% CI: 0.7-3.4). However, corresponding pooled negative and positive likelihood ratios independent of death rates were unsatisfactory for clinical usefulness in risk stratification.

**Conclusions:**

The presence of echocardiographic RV dysfunction or elevated natriuretic peptides is associated with short-term mortality in patients with pulmonary embolism without hemodynamic compromise. In contrast, the prognostic value of RV dilation on CT has yet to be validated in this population. As indicated both by positive and negative likelihood ratios the current prognostic value in clinical practice remains very limited.

## Introduction

A pulmonary embolism (PE) is a common and serious medical condition. The presence of shock or hemodynamic instability, defined as a systolic blood pressure of below 90 mm Hg or a drop of more than 40 mm Hg, is a clinical marker of high-risk patients who may benefit from early thrombolysis [1]. However, for those patients who are assessed to be at low or intermediate clinical risk but who are without hemodynamic compromise, this risk benefit is less clear. To refine therapeutic strategies in this subgroup, a more precise risk stratification is required with the hope that other patients who may benefit from thrombolytic therapy can be identified.

It is recognized that elevation in markers of myocardial ischemia and the presence of right ventricular dysfunction (RVD) have a negative prognostic impact, and they may define this intermediate risk group. The pathophysiology of RVD in a PE is thought to occur because of a sharp increase in RV afterload from both mechanical pulmonary arterial obstruction and serotonin-mediated pulmonary vasoconstriction. The resulting increases in wall stress and decreased oxygen supply cause RV myocardial ischemia, which in turn reduces left ventricular preload, cause of systemic hemodynamic instability.

Serum levels of cardiac troponin are specific for myocardial ischemia and infarction, and the prognostic impact of raised levels in PE was confirmed in a recent meta-analysis [2]. The prognostic value of other biological markers (for example, elevated natriuretic peptides), echocardiography, or computed tomography (CT) has been assessed only in small studies and recent meta-analyses [3,4]. We conducted this meta-analysis to assess the impact of echocardiographic, CT, and biological markers of RVD in PE on all-cause mortality within 3 months in low- or intermediate-risk patients who had no features of hemodynamic instability at presentation as well as to determine their prognostic value in terms of positive (PLR) and negative (NLR) likelihood ratios.

## Materials and methods

### Study objectives

The primary objective of this meta-analysis was to assess the prognostic value of these three RVD markers to predict mortality within 3 months in patients with acute PE. The secondary objective was to evaluate whether these markers are associated with short-term mortality resulting from PE or with serious adverse events (SAEs) in relation to RVD.

### Study endpoints

The primary endpoint was all-cause mortality. Secondary endpoints include death resulting from PE and SAE. Total death and death resulting from PE were adjudicated by the authors of the individual studies. Death resulting from PE was related to irreversible right heart failure or recurrent embolism at up to 90 days' follow-up. SAEs were the composite of death and any of the following adverse outcome events: shock, need for thrombolysis, nonfatal PE recurrence, cardiopulmonary resuscitation, mechanical ventilation, catecholamine administration, and surgical embolectomy.

### Search strategy

Database searches were performed in PubMed and the Cochrane database by using the combined medical subject headings for 'right ventricular dysfunction or right ventricular dilatation' with the exploded term 'acute pulmonary embolism' and by scanning references in retrieved articles and reviews. The retrieved studies were examined to exclude duplicate or overlapping data. Meeting abstracts were excluded because they could not provide adequately detailed data and their results might not be final.

### Study eligibility

Studies were eligible only if they evaluated the role of RVD on the primary endpoint and referred to subjects with non-high-risk PE. High-risk PE was defined as patients having shock or hypotension on hospital arrival. Inclusion criteria were (a) use of echocardiography, CT, or brain natriuretic peptide/pro-brain natriuretic peptide (BNP/proBNP) biomarkers for detecting RVD in patients with documented PE, (b) in consecutive patients identified either prospectively or retrospectively, (c) with a reported follow-up of at least 90 days, (d) for the primary endpoint of death or SAEs (or both) in relation to RVD, and (e) and studies that permitted the calculation of true positive (death with RVD), false positive (survival with RVD), true negative (survival without RVD), and false negative (death without RVD). Studies were excluded if they were performed (a) to test the efficacy or safety of thrombolysis or surgical embolectomy, (b) in patients without a definite diagnosis of PE, (c) with high-risk patients included, or (d) with fewer than 20 patients.

### Data extraction

The following information was extracted from each study: first author, year of publication, and journal; study population characteristics, including sample size; number of patients with documented PE; gender; mean age (and standard deviation); relative timing of RVD assessment; definitions of RVD on echocardiography and of RV dilatation on CT; technical characteristics of the BNP test and threshold, including type and brand of test used; and rate of short-term death or SAEs as previously defined according to RVD markers. Three investigators (GC, EC, and MaH) performed the data extraction independently. Discrepancies were solved by a consensus. The study was conducted in accordance with the guidelines of the Meta-analysis of Observational Studies in Epidemiology (MOOSE) [5]. In this meta-analysis, unlike in randomized controlled trials, no generally accepted lists of appropriate quality criteria for observational studies are available. Rather than producing a simple arbitrary quality score, specific quality aspects such as the following were used to assess the studies: control of confounding factors, minimization of selection bias with a clear description of inclusion and exclusion criteria, description of the baseline characteristics of the cohort, completeness of the follow-up, clear definition of study outcomes, relative timing of the RVD marker assessment after patient admission, and whether or not the investigator responsible for the RVD measurements was unaware of the patients' baseline characteristics or clinical course. Disagreements were solved by consensus.

### Data synthesis and statistical analysis

Pooled estimates for sensitivity, specificity, PLRs and NLRs, and odds ratio (OR) for the primary and secondary endpoints from individual studies were calculated by using a random-effects model as point estimates with 95% confidence intervals (CIs). Although sensitivity and specificity are well known as measures of diagnostic accuracy, their results may be influenced by the prevalence of disease in tested subjects. The PLR (the ratio between sensitivity and 1 - specificity) provides an estimate of the probability of a positive test in a patient with disease, and the NLR (the ratio between 1 - sensitivity and specificity) gives an estimate of the probability of a negative test among diseased subjects. Both likelihood ratios are roughly independent from prevalence rates, and there is consensus that a PLR of greater than 10 and an NLR of less than 0.1 provide reliable evidence of satisfactory diagnostic performance. While likelihood ratios are the recommended summary statistics for systematic reviews of diagnostic studies, predictive values may also be of interest for clinicians, even if these values vary widely in their dependence on disease prevalence.

Between-study statistical heterogeneity was assessed by using the Cochran Q chi-square test and the I^2 ^test. Separate analyses were performed on studies with the different RVD markers. Publication bias was assessed visually by examination of funnel plots. Statistical computations were performed with SPSS 11.0 (SPSS Inc., Chicago, IL, USA), Meta-Disc [6], and Review Manager 4.2, and significance testing was at the two-tailed 0.05 level.

## Results

### Description of studies

**Figure 1:**
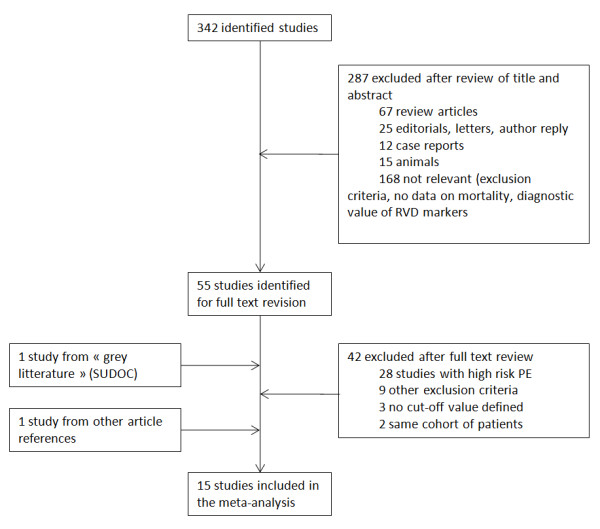
**Flow diagram for study selection**. PE, pulmonary embolism; RVD, right ventricular dysfunction; SUDOC, Système Universitaire de Documentation.

Searching performed until December 2009 allowed 15 studies to be included in this meta-analysis after the study selection described in Figure 1. Among those studies, 3 presented both echocardiographic and BNP results, leading to the analysis of 8 studies for echocardiographic markers of RVD (*n *= 1,249 patients) [7-14], 3 for CT markers (*n *= 503) [15-17], and 7 assessing natriuretic peptides (*n *= 582) [8,9,11,18-21]. The follow-up period varied between the in-hospital period and 3 months. All but one study were performed in a single center.

### Right ventricular dysfunction as assessed by echocardiography

**Figure 2:**
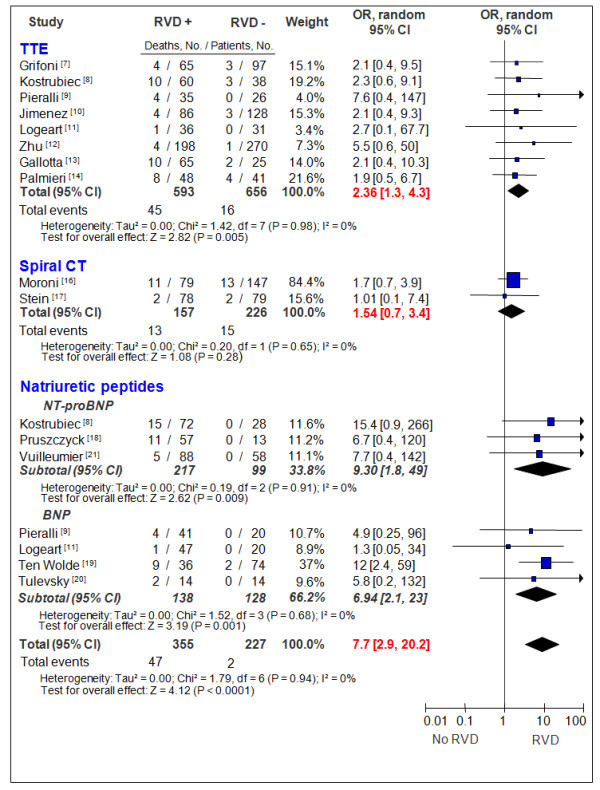
**Odds ratio for death, based on the presence or absence of right ventricular dysfunction markers in acute pulmonary embolism**. BNP, brain natriuretic peptide; CI, confidence interval; CT, computed tomography; NT-proBNP, N-terminal pro-brain natriuretic peptide; OR, odds ratio; RVD, right ventricular dysfunction; TTE, transthoracic echocardiography.

The echocardiographic criteria for each study are shown in Table 1, and all included a quantitative index of RV dilatation. The delay between the diagnosis of PE and performance of echocardiography varied between 1 and 48 hours. In these studies, the average mortality rate was 5% (range 1% to 13.5%), and the unadjusted OR of RVD in predicting death was 2.36 (95% CI 1.3 to 4.3), with no significant statistical heterogeneity (Figure 2). The pooled NLR of the RV dilation on echocardiography to predict mortality was unsatisfactory (0.62, 95% CI 0.41 to 0.92). The pooled sensitivity, specificity, negative (NPV) and positive (PPV) predictive values, and NLRs and PLRs are summarized in Table 2 for both the primary and secondary endpoints.

**Table 1:** Characteristics of included studies

Author	Study design	Patients, n	Delay	Primary outcome	SAE definition	Follow-up	Mortality, %	RVD definition	RVD, %	HI, n	Thrombolysis, n (%)	Age, years	Male, %	CHF, %	COPD, %
TTE															

Grifoni, *et al. *[7]	Prosp	162 (209^a ^)	<1 hour	Death	Clinical worsening, death	Hospital	4	1 in A1, A2, B, G, H1	40	0 (47 excluded)	10 (5%)	65 ± 15	40	14	25
Kostrubiec, *et al. *[8]	Prosp	98	<24 hours	Death	Death, vasopressor, thrombolysis, CPR	40 days	13	A9 + C or G + H1	60	0	5 (5%)	63 ± 18	38	17	7
Pieralli, *et al. *[9]	Prosp	61	<1 hour	Death	Death, PE recurrence, HI	Hospital	6.5	1 in A1, A2, B, C, G, H2	57	0	6 (10%)	75 ± 14	26	0	10
Jimenez, *et al. *[10]	Prosp	214	<48 hours	Death	Not studied	30 days	3	1 in A1, A2,C,F	40	0	NA	NA	49	11.7	13
Logeart, *et al. *[11]	Prosp	67	<19 hours	Death	Death, thrombolysis, HI	Hospital	1.5	2 in A3, B, C, D2, F	54	0	6 (9%)	64	60	0	NA
Zhu, *et al. *[12]	Prosp	468 (520^a ^)	NA	SAE	Death, thrombolysis, CPR, MV, embolectomy	14 days	1	2 in : A2 or A6, C, D3, F	42	0 (52 excluded)	NA	57 ± 14	62	NA	8
Gallota, *et al. *[13]	Prosp	90	<1 hour	SAE	Death, HI	Hospital	13	1 in A5, B	72	0	NA	67 ± 18	28	44	11
Palmieri, *et al. *[14]	Prosp	89	Admission	SAE	Death, HI	Hospital	13.5	A4 + B + C	54	0	NA	63 ± 15	27	NA	10

Spiral CT															

van der Meer, *et al. *[15]	Retro	120	NA	Death (PE)	Not studied	3 months	15	dRV/dLV >1	57.5	0	0	59 ± 18	46	NA	NA
Moroni [16]	Retro	226	NA	Death	Not studied	3 months	10.6	dRV/dLV >1	35	0	0	67 ± 17	50	14	6.5
Stein, *et al. *[17]	Retro	157	NA	Death	Not studied	30 days	2.5	dRV/dLV >1	50	0	2 (1.3%)	56 ± 17	41	0	0

NT-proBNP															

Kostrubiec, *et al. *[8]	Prosp	100	Admission	Death	Death, thrombolysis, CPR, embolectomy, vasopressors	40 days	15	>600 pg/mL	39	0	5	62 ± 18	35	17	7
Pruszczyk, *et al. *[18]	Prosp	70	Admission	Death	Death, thrombolysis, CPR, embolectomy, vasopressors	Hospital	15.7	NA	83.5	0 (9 excluded)	8	63 ± 17	37	NA	NA
Vuilleumier, *et al. *[21]	Prosp	146	Admission	Death	-	3 months	3.4	300 pg/mL	60	0	0	NA	42	NA	5

BNP															

Pieralli, *et al. *[9]	Prosp	61	Admission (<1 hour)	Death	Death, HI, PE recurrence	Hospital	6.5	>100 pg/mL	70	0	6	75 ± 14	26	Excluded	10
Logeart, *et al. *[11]	Prosp	67	Admission	Death	Death, thrombolysis, CPR, vasopressors	Hospital	1.5	>527 pg/mL	67	0	6	64 ± 16	60	Excluded	NA
ten Wolde, *et al. *[19]	Prosp	110	Admission	Death	Not studied	3 months	8.2	>21.7 pmol/L	33	0	NA	58 ± 18	NA	NA	NA
Tulevski, *et al. *[20]	Prosp	28	Admission (<1 hour)	Death	Not studied	90 days	7.1	>10 pmol/L	50	0	NA	53 ± 18	43	Excluded	0

^a^Before exclusion of patients with high-risk pulmonary embolism (PE). Right ventricular dysfunction (RVD) definition: (A1) end diastolic right ventricular diameter (EDRVD) of greater than 30 mm; (A2) EDRVD/left ventricular diameter (LVD) of greater than 1 in four-chamber view; (A3) EDRVD/LVD of greater than 0.7 in four-chamber view; (A4) EDRVD/LVD of greater than 0.9 in four-chamber view; (A5) EDRVD of greater than 15 mm/m^2 ^body surface area (BSA); (A6) EDRVD/LVD of greater than 0.6 in four-chamber view; (A7) EDRVD of greater than 4.5 cm in four-chamber view; (A8) right ventricular end diastolic area (RVEDA) greater than left ventricular area (LVA) in four-chamber view; (A9) EDRVD/LVD of greater than 0.6 parasternal long axis; (B) paradoxal septal motion; (C) right ventricular free wall hypokinesia; (D1) tricuspid valve regurgitation (jet velocity of greater than 2.5 m/s); (D2) tricuspid valve regurgitation (jet velocity of greater than 2.7 m/s); (D3) tricuspid valve regurgitation (jet velocity of greater than 2.8 m/s); (E) thrombi in right chambers; (F) loss of inspiratory collapse of the inferior vena cava; (G) tricuspid valve pressure gradient (TVPG) of greater than 30 mm Hg; (H1) Doppler pulmonary acceleration time of less than 80 ms; (H2) Doppler pulmonary acceleration time of less than 90 ms; (I) right pulmonary artery dilatation of greater than 12 mm/m^2 ^BSA. CHF, chronic heart failure; COPD, chronic obstructive pulmonary disease; CPR, cardiopulmonary resuscitation; d, diameter; HI, hemodynamic instability; hospital, in-hospital follow-up; LV, left ventricle; MV, mechanical ventilation; NA, not applicable; prosp, prospective; retro, retrospective; RV, right ventricle; SAE, serious adverse event; TTE, transthoracic echocardiography.

**Table 2:** Pooled summary results of the prognostic value of right ventricular dysfunction markers

	Number of patients	Number of studies	Odds ratio	Sensitivity, %	Specificity, %	PLR	NLR	PPV, %	NPV, %
TTE Death all-cause	1,249	8	2.36 (1.3-4.3)	74 (61-84)	54 (51-56)	1.4 (1.2-1.6)	0.62 (0.41-0.92)	7.6 (5.6-10)	97.6 (96-98.6)
TTE PE-related death	781	7	4.44 (1.75-11.3)	92 (78-98)	51 (48-55)	1.65 (1.4-2)	0.36 (0.2-0.8)	8.4 (6-11)	99 (98-100)
TTE SAE	1,035	7	4.03 (2.76-5.9)	77 (71-83)	58 (54-61)	1.73 (1.5-1.9)	0.46 (0.3-0.6)	30 (26-34)	92 (89-94)
CT Death all-cause	383	2	1.54 (0.7-3.4)	46 (27-66)	59 (54-64)	1.3 (0.4-2)	0.8 (0.6-1.2)	8.3 (4.5-14)	93 (89-96)
CT PE-related death	277	2	2.17 (0.06-79)	87.5 (47-100)	48 (42-54)	1.2 (0.25-6)	0.51 (0.007-36)	5 (2-9)	99 (96-100)
CT SAE	0	0	-	-	-	-	-	-	-
BNP-ProBNP Death all-cause	582	7	7.7 (2.9-20.2)	96 (86-100)	42 (38-46)	1.5 (1.2-1.9)	0.26 (0.1-0.6)	13 (10-17)	99 (97-100)
BNP-ProBNP PE-related death	436	6	6.4 (2-20)	97 (84-100)	42 (37-47)	1.5 (1.2-1.9)	0.3 (0.1-0.7)	12 (8-16)	97 (84-100)
BNP-ProBNP SAE	228	3	15.6 (3-82)	100 (91-100)	36 (30-44)	1.5 (1.3-1.7)	0.01 (0.02-0.5)	26 (19-33)	100 (91-100)

Values are presented as numbers or as percentages (ranges). BNP-ProBNP, brain natriuretic peptide-N-terminal pro-brain natriuretic peptide; CT, computed tomography; NLR, negative likelihood ratio; NPV, negative predictive value; PE, pulmonary embolism; PLR, positive likelihood ratio; PPV, positive predictive value; SAE, serious adverse event; TTE, transthoracic echocardiography.

### Right ventricular dysfunction as assessed by computed tomography

RV dilatation criteria were the same in the three included studies: right-to-left ventricular minor axis dimension ratio of greater than 1, measured at the widest points between the inner surface of the free wall and the surface of the interventricular septum (Table 1). All-cause mortality was not given in one study [15]. In the two other studies, the average mortality rate was 7.3% (range 2.5% to 15%); however, RVD was not associated with the death (Figure 2). The pooled sensitivity, specificity, NPVs and PPVs, and NLRs and PLRs are summarized in Table 2 for both the primary and secondary endpoints.

### Right ventricular dysfunction as assessed by BNP/NT-proBNP elevation

Of the seven included studies, only one used a predefined cutoff value for N-terminal pro-brain natriuretic peptide (NT-proBNP) [21]. Other studies used a cutoff value derived from receiver operating characteristic curve construction to determine the best threshold able to predict complicated PE. Plasma concentrations of NT-proBNP (enhanced chemiluminescence immunoassay; Roche, Basel, Switzerland [8,18,21]) and BNP (fluorescence immunoassay: Triage; Biosite Incorporated, San Diego, CA, USA [9,11], and immunoradiometric assay: Shionoria; Shionogi & Co., Ltd., Osaka, Japan [19,20]) were quantitatively assessed by using autoanalyzers. In five studies [8,9,11,19,20], clinicians were blind to the results of natriuretic peptides, and in the two remaining studies [18,21], this information was not presented. Overall, the average mortality rate was 8.1% (range 1.5% to 15.7%), and the unadjusted OR of elevated natriuretic peptides in predicting death was 7.7 (95% CI 2.9 to 20.2), with no significant statistical heterogeneity between studies and between studies using BNP and NT-proBNP (Figure 2). However, the NLR remains nonoptimal (0.26, 95% CI 0.1 to 0.6). The pooled sensitivity, specificity, NPVs and PPVs, and NLRs and PLRs are summarized in Table 2 for both the primary and secondary endpoints.

## Discussion

This analysis indicates that RVD as assessed by echocardiography or elevated BNP/proBNP levels can help to identify patients with PE without hemodynamic compromise at increased risk of short-term death and adverse outcomes. In contrast, CT markers, including RV dilation, were unable to identify a similar risk group. However, given the limitations for each marker, their predictive ability should be treated with some caution, as detailed below.

### Right ventricular dysfunction as assessed by echocardiography

Although echocardiographic markers had an excellent NPV and therefore should be able to predict a good outcome efficiently (pooled NPV to predict overall mortality: 98%, 95% CI 96% to 99%), this statistic is influenced by the prevalence of death. As overall death rates were low in this population of intermediate- and low-risk patients (5%, range 1% to 13.5%), the NLR would be a better assessment of its usefulness. The pooled NLR of the RV dilation on echocardiography to predict mortality was unsatisfactory (0.62, 95% CI 0.41 to 0.92). Thus, the prognostic value of the absence of RV dilation on echocardiography remains uncertain. More importantly, the definition of RVD differed greatly among the studies, and patients with chronic obstructive pulmonary disease were not excluded [10]. In addition, differentiation of chronic and acute RV overload would be difficult using standardized criteria (RV free wall thickness of greater than 5 mm or tricuspid valve regurgitation jet velocity of greater than 3.7 m/s or both). Other limitations include publication bias, despite an exhaustive database search, as demonstrated in the funnel plot: small negative or weakly positive studies are not published (data not shown). Sensitivity analysis showed a significant loss in predictive power of these markers after exclusion of the small studies. However, the prognosis impact of these markers remained statistically significant (data not shown). Finally, when common confounding factors were controlled for by a multivariate analysis (performed with the data from five studies [8,10,12-14]), this effect was apparent in only one study [12], thus diminishing the importance of this marker further.

### Right ventricular dysfunction as assessed by computed tomography

Although CT scanning has a high availability and plays a central role in the diagnosis of a PE, a role in determining short-term prognosis is unclear. Detection of RV dilation has been reported to be useful [22]. However, our analysis demonstrated this to be of limited prognostic importance and is in agreement with the recent meta-analysis by Sanchez and colleagues [4], who found no statistically significant relation between RV dilatation on CT and mortality among patients with non-high-risk PE.

In contrast to echocardiography, CT markers of RV dilatation were homogeneous between studies. However, recent data have shown that measurements made on the four-chamber view with electrocardiogram gating are more reliable than traditional measurements made on the minor axis.

Our analysis had some limitations. For example, the numbers of patients in this subgroup were small, the majority of the studies were retrospective, and the clinical presentation of patients included in studies is not widely reported. Hence, any conclusions about the usefulness of this marker must be treated with some caution, and in the future, larger clinical studies and standardized definitions of RV dilation will be required in this patient subset.

### Right ventricular dysfunction as assessed by BNP or NT-proBNP elevation

The availability of biomarkers like BNP or proBNP enables early identification of patients with RVD and can contribute to risk stratification; this is potentially important, especially when echocardiography assessment is not available. We confirmed that BNP or proBNP levels identified patients at higher risk of poor outcomes, but because they had a low specificity and consequently low PPV, their clinical use to identify those at risk of mortality in this population may be limited.

Additionally, despite apparently superior diagnostic performance, a direct comparison with echocardiographic techniques for identifying those at risk of death may be misleading as these biomarker-based studies appeared to enroll populations of a higher average mortality in comparison with echocardiography (8.1%, range 1.5% to 15.7% versus 5%, range 1% to 13.5%; *P *< 0.01). There was no evidence of publication bias (funnel plot not shown). Multivariate analysis showed a stability of the prognosis value of natriuretic peptides [18,19,21], with the exception of one study [8].

Our analysis was limited by the small overall sample size (*n *= 436), as demonstrated by the wide CI of the calculated point estimates (Figure 2); as for echocardiography, despite an excellent NPV, the NLR remains nonoptimal (0.26, 95% CI 0.1 to 0.6). Moreover, in most of the studies, the cut points for BNP assays were not predefined but were derived from receiver operating characteristic curve construction to determine the best threshold able to predict a complicated PE. Finally, BNP and NT-proBNP are nonspecific markers of wall ventricular stress and can be elevated in clinical conditions other than PE, such as chronic heart failure (CHF). The fact that only three of the seven studies [9,11,20] excluded CHF patients could be a potential source of bias as the majority of patients with CHF have elevated BNP levels and are at higher risk of mortality during PE in comparison with the population without CHF.

## Conclusions

The presence of echocardiographic RVD or elevated natriuretic peptides is associated with short-term mortality in patients with intermediate- and low-risk PE. In contrast, the prognostic value of RV dilation on CT has yet to be validated in this population. However, the clinical utility of these markers is at risk of being overstated given the number of limitations previously mentioned. Based on the present analysis, the prognosis performance of markers for RVD to predict death or adverse outcomes is too low to be useful in routine practice for patient management. Only large prospective studies will be able to show whether the presence of such markers or their combination, associated with markers of myocardial ischemia, might justify initial aggressive treatment in some subsets of patients.

## Key messages

• Echocardiographic right ventricular (RV) dysfunction or elevated natriuretic peptides are associated with short-term mortality in patients with pulmonary embolism without hemodynamic compromise.

• The prognostic value of RV dilation on computed tomography has yet to be validated in this population.

• As indicated by both positive and negative likelihood ratios, the current prognostic value of RV dysfunction markers remains very limited in clinical practice.

## Abbreviations

BNP: brain natriuretic peptide; CHF: chronic heart failure; CI: confidence interval; CT: computed tomography; NLR: negative likelihood ratio; NPV: negative predictive value; NT-proBNP: N-terminal pro-brain natriuretic peptide; OR: odds ratio; PE: pulmonary embolism; PLR: positive likelihood ratio; PPV: positive predictive value; RV: right ventricular; RVD: right ventricular dysfunction; SAE: serious adverse event.

## Competing interests

The authors declare that they have no competing interests.

## Authors' contributions

GC, EC, and MiH conceived of and designed the research. MaH performed statistical analysis. All authors analyzed and interpreted the data and helped to draft the manuscript and revise it for important intellectual content. All authors read and approved the final manuscript.
